# *Taenia crassiceps*-Excreted/Secreted Products Induce a Defined MicroRNA Profile that Modulates Inflammatory Properties of Macrophages

**DOI:** 10.1155/2019/2946713

**Published:** 2019-05-14

**Authors:** Diana Martínez-Saucedo, Juan de Dios Ruíz-Rosado, César Terrazas, Blanca E. Callejas, Abhay R. Satoskar, Santiago Partida-Sánchez, Luis I. Terrazas

**Affiliations:** ^1^Unidad de Biomedicina, Facultad de Estudios Superiores Iztacala, Universidad Nacional Autónoma de México, Estado de México, Mexico; ^2^Center for Microbial Pathogenesis, The Research Institute at Nationwide Children's Hospital, Columbus, Ohio, USA; ^3^Department of Pathology, The Ohio State University, Columbus, OH, USA; ^4^Laboratorio Nacional en Salud, Facultad de Estudios Superiores Iztacala, Universidad Nacional Autónoma de México, Estado de México, Mexico

## Abstract

Helminth parasites modulate immune responses in their host to prevent their elimination and to establish chronic infections. Our previous studies indicate that *Taenia crassiceps*-excreted/secreted antigens (TcES) downregulate inflammatory responses in rodent models of autoimmune diseases, by promoting the generation of alternatively activated-like macrophages (M2) *in vivo*. However, the molecular mechanisms triggered by TcES that modulate macrophage polarization and inflammatory response remain unclear. Here, we found that, while TcES reduced the production of inflammatory cytokines (IL-6, IL-12, and TNF*α*), they increased the release of IL-10 in LPS-induced bone marrow-derived macrophages (BMDM). However, TcES alone or in combination with LPS or IL-4 failed to increase the production of the canonical M1 or M2 markers in BMDM. To further define the anti-inflammatory effect of TcES in the response of LPS-stimulated macrophages, we performed transcriptomic array analyses of mRNA and microRNA to evaluate their levels. Although the addition of TcES to LPS-stimulated BMDM induced modest changes in the inflammatory mRNA profile, it induced the production of mRNAs associated with the activation of different receptors, phagocytosis, and M2-like phenotype. Moreover, we found that TcES induced upregulation of specific microRNAs, including miR-125a-5p, miR-762, and miR-484, which are predicted to target canonical inflammatory molecules and pathways in LPS-induced BMDM. These results suggest that TcES can modulate proinflammatory responses in macrophages by inducing regulatory posttranscriptional mechanisms and hence reduce detrimental outcomes in hosts running with inflammatory diseases.

## 1. Introduction

Helminth infections induce polarized T_H_2-type biased immune responses that play a role in parasite expulsion, tissue repair, and regulation of unrelated inflammatory and autoimmune responses in the host [1–3]. The striking ability of helminth parasites in conferring protection from diseases of immune dysregulation has increased the attention into the immunomodulatory mechanisms evoked by these pathogens. Previous studies in our laboratory, using a murine model of cysticercosis, demonstrated that chronic infection with the helminth *Taenia crassiceps* or administration of its excreted/secreted products (TcES) ameliorates the development of experimental ulcerative colitis, autoimmune encephalomyelitis (EAE), and type 1 diabetes [4–8]. The ability of *T. crassiceps* and TcES to counteract these inflammatory responses was demonstrated to be dependent on a population of macrophages that produced markers of alternative activation (M2), such as PD-L2, IL-4R*α*, MR, IL-10, ARG1, YM1, and FIZZ1 [9].

Macrophages can be activated towards an M2 phenotype after being stimulated with IL-4 produced by T_H_2 lymphocytes during parasitic infections or exposure to allergens [10, 11]. In contrast, released IFN-*γ* and pathogen or danger-associated molecular patterns (PAMPs or DAMPs) during infections or tissue injury, respectively, promote classical (M1) activation in macrophages [1, 12]. Although a crucial role for *T. crassiceps-*induced M2 macrophages in regulating detrimental autoimmune and inflammatory responses has been demonstrated [3], the transcriptional events elicited by TcES that modulate macrophage activation have not been elucidated.

Helminth infections and/or their antigens can trigger the levels of microRNAs to modulate inflammatory responses in the host [13–15]. MicroRNAs are small noncoding RNAs that regulate cell functions posttranscriptionally through direct binding to the 3′-UTR (untranslated region) of target messenger RNAs (mRNAs), resulting in the destabilization of mRNAs and repression of translation [16]. Recently, microRNAs have been associated with helminth-induced M2 macrophages *in vitro* and *in vivo*. For instance, Rückerl et al. reported that macrophages obtained during acute (3 weeks) *Brugia malayi* infection induced microRNAs associated with M2 macrophages, such as miR-199-5p, miR-378-3p, and miR-125b-5p [15]. In addition, Guo and Zheng identified distinct microRNAs, including miR-146a-5p, miR-155-5p, miR-21a-5p, miR-146b-5p, miR-99b-3p, miR-125a-5p, and miR-378, in RAW 264.7 macrophages cocultured with metacestodes of *Echinococcus multilocularis*. In these studies, the authors suggested a role for these microRNAs in targeting important inflammatory mRNAs (*Tnf*, *Il1a*, *Il6*, *Il12a*, *Il12b*, *Ccl22*, and *Ccl18* mRNA) [14]. Thus, microRNAs may be a key mechanism elicited by helminths in the regulation of inflammatory responses in the host.

Although we have previously demonstrated a role for the TcES in preventing STAT1 phosphorylation in inflammatory macrophages [17], the influence of TcES in macrophage polarization and the transcriptional pathways regulating this process remain unknown. Here, we determined the effect of TcES alone or in combination with LPS or IL-4, in the regulation of multiple mRNA transcripts and microRNAs induced in macrophages. Our results indicate that TcES decreased the production of inflammatory cytokines (IL-12, TNF*α*, and IL-6) in LPS-induced macrophages but has a limited role in inducing directly the production of M1- and/or M2-associated molecules. The immune-modulatory ability of TcES was further associated with increased levels of specific microRNAs, which are predicted to target, according to our bioinformatic analysis, numerous inflammatory mRNAs involved in the TNF and NF-*κ*B signaling pathways. These findings suggest a role for TcES in modulating the transcriptional profile of macrophages via altering their microRNA profile and, consequently, the inflammatory properties of these immune cells.

## 2. Materials and Methods

### 2.1. Ethics Statement

All experiments in this study were performed according to the guidelines for the Care and Use of Laboratory Animals adopted by the US National Institutes of Health. The Institutional Animal Care and Use Committee (IACUC) at the Research Institute at Nationwide Children's Hospital and the Ohio State University approved all protocols.

### 2.2. Mice

Adult 6- to 8-week female BALB/c mice were purchased from The Jackson Laboratory. All animals were maintained in a pathogen-free environment and established as breeding colonies in the Transgenic Mouse Facility at the Research Institute at Nationwide Children's Hospital or in specific pathogen-free conditions at the Ohio State University Laboratory Animal Resources. The mice were housed in sterilized polycarbonate cages with basic filter top caging containing pine wood shavings and were offered mouse ration and water *ad libitum*. The cages were held in Isolation and Containment cubicles (Britz and Co., Wheatland, WY).

### 2.3. Parasites and TcES

Metacestodes of *T. crassiceps* (ORF strain) were harvested under sterile conditions from the peritoneal cavity of female BALB/c mice after 8-10 weeks of intraperitoneal (i.p.) infection. The cysticerci were washed four times in physiological saline solution prior to maintaining them in culture with a sterile saline solution at 37°C for 24 h. The supernatant was recovered and centrifuged for 10 min at 1000 g. The heavy fraction of TcES was concentrated using the 50 kDa Amicon Ultra Filter (Millipore), 30 min at 1000 g. Protease inhibitors were added to the >50 kDa fraction, and samples were stored at -70°C until further use.

### 2.4. Bone Marrow-Derived Macrophages (BMDM)

To generate bone marrow-derived macrophages (BMDM), we followed the protocol previously described [18]. Briefly, bone marrow cells were obtained by flushing femurs and tibias from BALB/c mice with a sterile saline solution. The isolated cells were plated in 100 mm Petri dishes at 1 × 10^6^ cells/mL in Dulbecco's modified Eagle's media (DMEM, Mediatech, Herndon, VA), supplemented with 10% heat-inactivated fetal bovine serum (FBS) (Life Technologies, Grand Island, NY), 1% penicillin/streptomycin, 1% glutamine, and 20 ng/mL of macrophage colony-stimulating factor (M-CSF, BioLegend). On day 7, the cells were harvested, washed, counted, and replated in culture media (without M-CSF) at a density of 2 × 10^6^ cells/well (12-well plate, Falcon polystyrene). BMDM were incubated with either TcES (25 *μ*g/mL), *Escherichia coli* LPS (1 mg/mL, Sigma-Aldrich), interleukin-4 (20 ng/mL), TcES+LPS or TcES+IL-4. After 4 and 24 h poststimulation, BMDM were harvested for flow cytometric and transcriptomic analysis. The supernatants were recovered for cytokine detection by ELISA.

### 2.5. Flow Cytometric Analysis

Flow cytometric analysis was performed as previously described [19]. Briefly, harvested BMDM were incubated in 1 *μ*g/mL of anti-mouse Fc receptor antibody in 100 mL PBS containing 0.5% BSA plus 0.02% NaN_3_ (FACS buffer) for 15 min on ice. Subsequently, single-cell suspensions were stained for 15 min at 4°C with blue-fluorescent reactive dye, L23105 (Life Technologies) to discriminate dead cells. After washing, 1-3 × 10^6^ cells were surface-stained in FACS buffer for 15 min at 4°C with antibodies recognizing CD11b (Alexa Fluor 700, BioLegend), F4/80 (Brilliant Violet 785, BioLegend), CD86 (Brilliant Violet 421, BioLegend), PD-L1 (PE-Cy7, BioLegend), and PD-L2 (PE, BioLegend). Surface-stained cells were washed three times with FACS buffer and treated with Fix/Perm reagent according to the protocol of the cytofix/cytoperm kit (BD Biosciences, San Jose, CA, USA). The cells were intracellularly stained in FACS buffer containing anti-Nos2 (PE, eBiosciences) and anti-h/m arginase 1 (APC, R&D systems) for 30 min at 4°C and further collected on an LSR II cytofluorometer (BD, Franklin Lakes, NJ). Stained cells were gated according to size (SSC-A) and forward scatter (FSC-A) to eliminate debris. Doublets were excluded from the analysis by using forward scatter height (FSC-H) and FSC-A. Data analysis was performed using FlowJo Software (FlowJo, LLC).

### 2.6. Cytokine Assay

Supernatants from cell cultures of stimulated macrophages were recovered at 4 and 24 h poststimulation, and the levels of the cytokines IL-10, IL-6, TNF*α*, and IL-12 were measured by ELISA according to the manufacturer's instructions (PeproTech).

### 2.7. RNA Extraction and Arrays

Total RNA was extracted from BMDM stimulated with LPS (M_LPS_), TcES (M_TcES_), LPS+TcES (M_TcES+LPS_), or culture media (M_0_) using QIAzol reagent (QIAGEN), according to the manufacturer's specifications, and stored at -80°C. Next, RNA was purified following the miRNeasy kit protocol (QIAGEN). RNA concentration and integrity were determined using a NanoDrop™ spectrophotometer (Thermo Scientific, Wilmington, DE) and Agilent Bioanalyzer 2100, respectively. For transcriptomic analysis, 50 ng/*μ*L of RNA was used for the nCounter Inflammation Panel (NanoString mRNAs) and the nCounter miRNA Assay set (microRNAs). Both mRNA and microRNA arrays were performed following the manufacturer's instructions at the Genomics Shared Resource, OSU. Data analysis for the nCounter Inflammatory Panel (mRNA) and for the nCounter miRNA Assay set was conducted using the nSolver™ Analysis Software according to the manufacturer. For the nCounter Inflammatory Panel (mRNA), we normalized using the normalization factor and subtracted the background (mean of negative controls ± 2 standard deviations). Next, we normalized using the geometric mean of housekeeping genes as reported [20]. Then, using the normalized counts, we calculated the fold change (FC) by comparing M_TcES_, M_TcES+LPS_, and M_LPS_ to M_0_. For the nCounter miRNA Assay set, we first normalized using the normalization factor. The background was subtracted from the data using the mean of negative controls ± 2 standard deviations. Finally, we used the top 75 microRNAs [21]. The normalized counts were used to calculate the FC by comparing M_TcES_, M_TcES+LPS_, and M_LPS_ to M_0_. Of the 566 total probes measured in the assay, 183 and 236 microRNAs for 4 h and 24 h, respectively, were identified and used for analyzing significant changes in microRNA levels among samples. MultiExperiment Viewer (MeV) was used to generate heat maps, which represent log_2_-transformed data.

### 2.8. Real-Time PCR

TaqMan gene expression assays (Applied Biosystems) were used to quantify and/or validate the levels of mRNAs and microRNA transcripts. cDNA was generated from mRNAs, using a 15 *μ*L RT reaction consisting of 2.0 *μ*L of Buffer (10x), 0.8 *μ*L 100 mM dNTPs (100 mM), 1.0 *μ*L reverse transcriptase, 2.0 *μ*L of mRNA primer, and 1 *μ*g of total RNA. RT reaction was incubated for 30 min at 16°C, 30 min at 42°C, and 5 min at 85°C. For microRNA levels, a 15 *μ*L reaction was prepared with 2.0 *μ*L of buffer (10x), 0.2 *μ*L 100 mM dNTPs (100 mM), 1.0 *μ*L reverse transcriptase, 0.2 *μ*L RNAse inhibitor (20 U/*μ*L), 3.0 *μ*L of microRNA primer, and 100 ng of total RNA. RT reaction was incubated as mentioned before. For both mRNA and microRNAs, quadruplicate real-time PCR reactions were performed in the 7500 Real-Time PCR system. The amplification reaction mix was composed of 10 *μ*L of TaqMan Universal PCR Master Mix (2x), 1 *μ*L of the specific mRNA or microRNA probe, and 1 *μ*L of specific microRNA cDNA. The reactions were preincubated for 10 minutes at 95°C and amplified with 40 cycles consisting of 10 sec at 95°C, 40 sec at 60°C, and 5 sec at 72°C (fluorescence acquisition). To assess possible bias for reference RNA, we used 18S RNA, *Actb*, and *Gapdh* mRNAs. Relative quantification was calculated by 2^-ΔΔCt^. All mRNA and microRNA assays were tested for reproducibility and linearity (PCR efficiency was between 1.9 and 2.0 for all assays). All primers were purchased from Applied Biosystems. The primer sequences are shown in Table S1.

### 2.9. mRNA and MicroRNA Target Gene Prediction and Bioinformatics Analysis

Target mRNAs of differentially produced microRNAs were predicted using DIANA-TarBase database v6.0, which includes experimentally validated targets from the literature. To explore the potential biological function of the microRNAs' profile and their targets, DIANA-mirPath v2.0 (http://snf-515788.vm.okeanos.grnet.gr/) was used to perform enrichment analysis of microRNA's target mRNAs in the KEGG pathway and in GO terms [22].

### 2.10. Statistical Analysis

Data analyses were performed using GraphPad Prism 6 software. Statistical comparisons were performed by using Student's *t*-test. *p* values less than 0.05 were considered significant. Graphed data are presented as mean ± SD or SEM.

## 3. Results

### 3.1. TcES Reduces the Inflammatory Response of LPS-Induced BMDM

**Figure 1:**
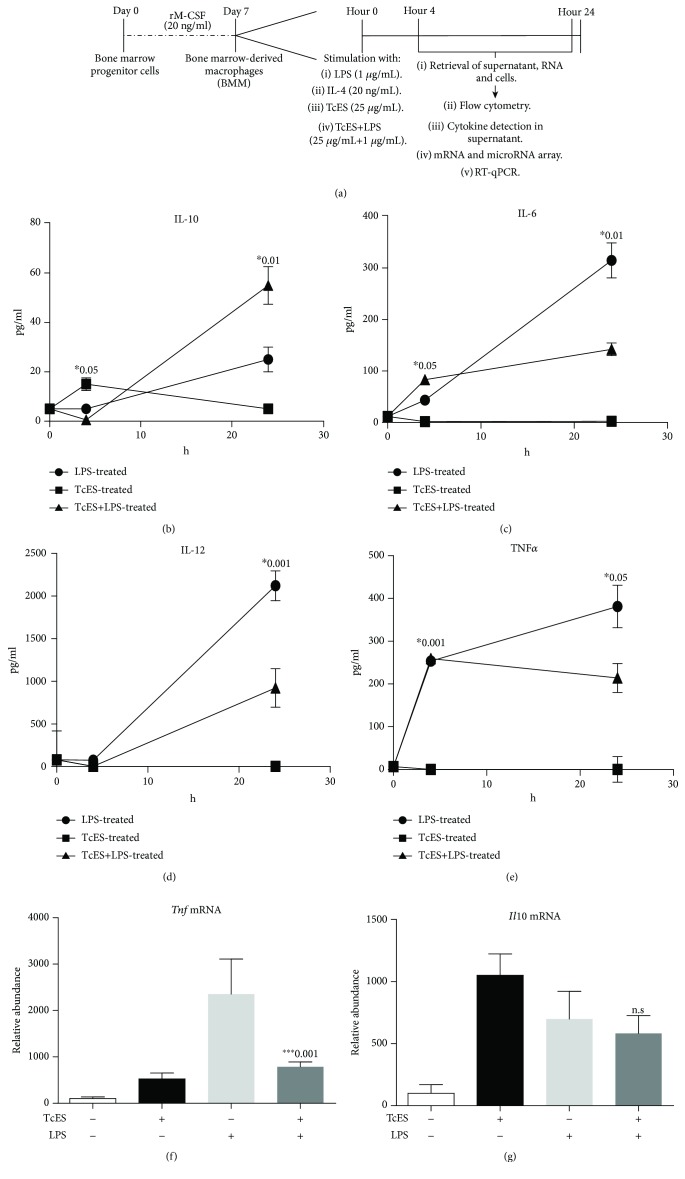
TcES regulates the production of inflammatory cytokines in LPS-induced macrophages. (a) Flow diagram of general experimental design. For differentiation of macrophages, bone marrow progenitor cells were cultured with rM-CSF at 37°C 5% CO_2_ for 7 days. On completion day, BMDM were washed and stimulated with the following stimuli: LPS (1 *μ*g/mL), TcES (25 ng/mL), or both for 4 or 24 hours. The supernatants, total RNA or cells were harvested for later procedures. (b) Kinetics levels of IL-10, (c) IL-6, (d) IL-12, and (e) TNF*α* in supernatants from stimulated BMDM. Evaluation of the levels of (f) *Tnf* and (g) *Il10* mRNA by RT-qPCR in groups of stimulated macrophages mentioned above (*n* = 6, 3 replicates condition). mRNA levels are represented as mean relative (±SD). Data are representative of 3 independent experiments using 3 replicates per stimuli. Significance was calculated using *t*-test. ^∗^ *p* < 0.01, ^∗∗^ *p* < 0.05, and ^∗∗∗^ *p* < 0.001.

Previously, we demonstrated the ability of TcES in reducing the development of inflammatory and autoimmune diseases in rodent models [4–8]. The effect of TcES in counteracting detrimental inflammatory responses *in vivo* is associated with the emergence of polarized macrophages towards an M2 phenotype [4, 5, 11]. Although studies in our laboratory indicate a role for TcES in blocking the IFN-*γ*/STAT1 signaling pathway in macrophages [17], the effect of TcES in inducing directly M2 macrophages remains to be elucidated. To define the macrophage profile elicited by TcES, we first determined the levels of the inflammatory cytokines IL-12, IL-6, TNF*α*, and IL-10 in cultures from BMDM. The cells were stimulated (Figure 1(a)) for 4 h or 24 h with TcES (henceforth M_TcES_), *E. coli* lipopolysaccharide (M_LPS_), interleukin-4 (M_IL-4_), TcES+LPS (M_TcES+LPS_), TcES+IL-4 (M_TcES+IL-4_), or PBS (M_0_). Supernatants obtained from M_TcES_ displayed higher levels of IL-10 and deficient levels of inflammatory cytokines (IL-6 and TNF*α*) compared to all the groups at 4 h poststimulus (Figures 1(b) and 1(e)). However, IL-10 production by M_TcES_ did not continue at 24 h. Interestingly, we found that exposure of macrophages to TcES and stimulated with LPS (M_TcES+LPS_) significantly reduced the production of IL-12, IL-6, and TNF*α* compared to those in M_LPS_ at 24 h (Figures 1(c) and 1(e)). Increased IL-10 levels were observed in supernatants from M_TcES+LPS_ compared with all groups at 24 h (Figure 1(b)). A similar trend was identified in the levels of the mRNA for *Tnf* at 24 h poststimulus (Figure 1(f)), whereas levels of *Il10* mRNA were similar between all groups at 24 h (Figure 1(g)). Our results suggest that TcES play a role in downregulating the production of proinflammatory cytokines in LPS-induced BMDM, by increasing the production of a regulatory cytokine.

**Figure 2:**
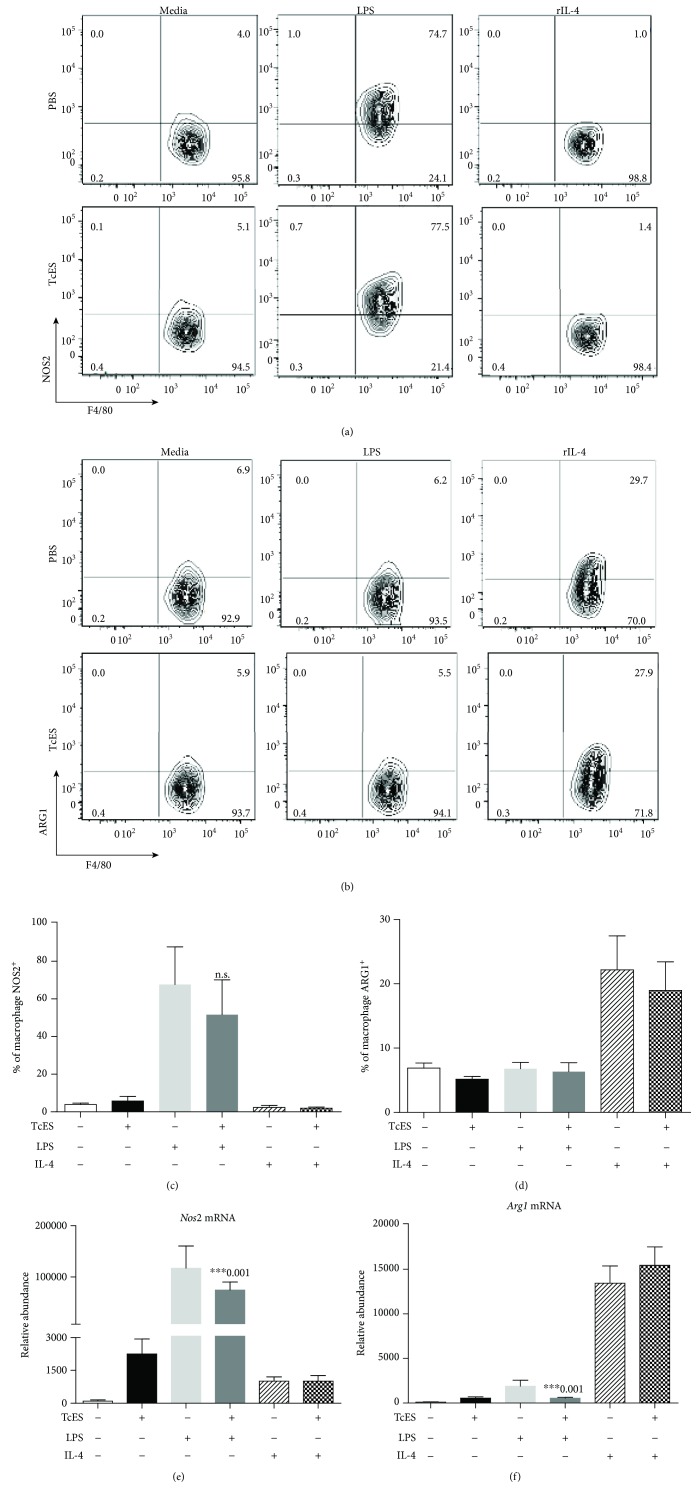
TcES do not modify the production of canonical M1/M2 macrophage markers. Representative dot plots, obtained by flow cytometry, of (a) F4/80^+^NOS2^+^ and (b) F4/80^+^ARG1^+^ BMDM, after 24 h poststimulus with one of the following stimuli: LPS (1 *μ*g/mL), TcES (25 ng/mL), IL-4 (20 ng/mL), TcES+LPS, TcES+IL-4, or PBS. Bar graphs representing the percentage of (c) F4/80^+^NOS2^+^ and (d) F4/80^+^ARG1^+^ BMDM at 24 h poststimulus. (e) Evaluation of the levels of *Nos2* and (f) *Arg1* mRNA by RT-qPCR in BMDM stimulated for 24 h (*n* = 6, 3 replicates condition). mRNA levels are represented as mean relative (±SD). Data are shown as a representative of two independent experiments. Significance was calculated using *t*-test. ^∗^ *p* < 0.01, ^∗∗^ *p* < 0.05, and ^∗∗∗^ *p* < 0.001.

To gain insight in the phenotypic profile induced by TcES in macrophages, we used flow cytometry technique to determine the production of intracellular nitric oxide synthase (NOS2), and arginase-1 (ARG1), as the conventional markers for M1 and M2 profiles, respectively, in BMDM. Our results showed that while M_LPS_ and M_IL-4_ presented increased percentages of NOS2^+^ and ARG1^+^ macrophages, respectively, M_TcES_ displayed limited production of these molecules (Figures 2(a) and 2(d)). Additionally, similar percentages of NOS2^+^ BMDM were found between M_TcES+LPS_ and M_LPS_, and comparable ARG1^+^ BMDM were observed when analyzing M_TcES+IL-4_ versus M_IL-4_ (Figures 2(a) and 2(d)). Levels of mRNA *Arg1* by RT-qPCR showed similar trends as the flow cytometric analysis (Figure 2(f)). While the levels of *Nos2* mRNA were upregulated in M_TcES+LPS_ compared to M_0_ but significantly reduced compared to M_LPS_ (Figure 2(e)). These data suggest that the stimulus with TcES, either alone or in combination with LPS or IL-4, has a limited role in inducing the production of canonical M1 or M2 markers. Nevertheless, these antigens play a role in downregulating the proinflammatory response to LPS in BMDM.

### 3.2. TcES Modify the Proinflammatory mRNA Profile of LPS-Induced BMDM

**Figure 3:**
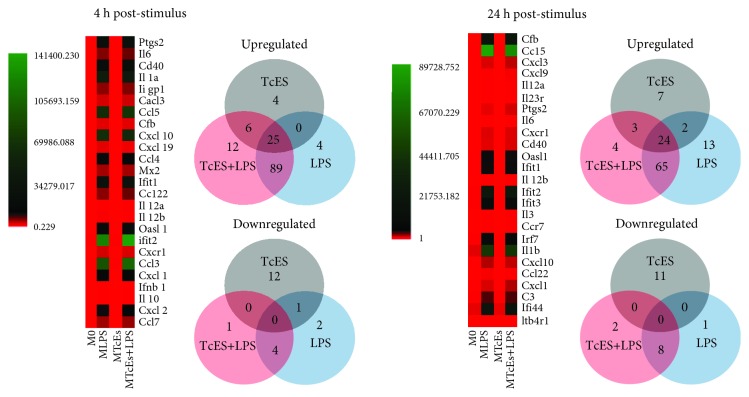
Top 25 upregulated mRNAs in stimulated BMDM. Heat maps show mRNA levels among M_TcES_, M_TcES+LPS_, and M_LPS_ at 4 and 24 h poststimulus. Each row represents mRNA levels, and each column represents a specific sample. The color scale illustrates the relative levels of mRNA: green, increased production; red, decreased production; and black, mean value. Venn diagrams show the unique and overlapping mRNA transcripts among the samples.

**Figure 4:**
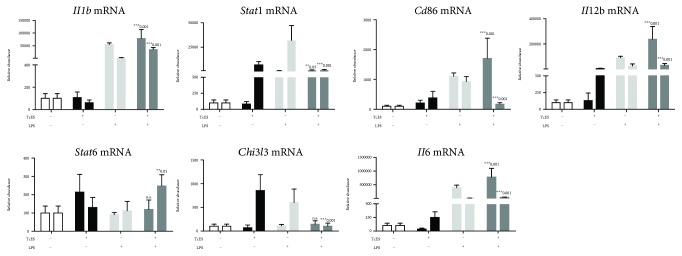
Validation and identification of mRNAs in stimulated BMDM. Macrophages were cultured in the presence of TcES (25 *μ*g/mL), LPS (1 *μ*g/mL), or both TcES+LPS for 4 (left bar) and 24 h (right bar) poststimulation. Relative levels of selected mRNA were determined by TaqMan mRNA assays after normalization with 18S RNA. The levels of mRNA are represented as fold change relative to the PBS-treated group (FC ± SD). Data shown are representative of two independent experiments (*n* = 6, 3 replicates condition). Significance was calculated using *t*-test. ^∗^ *p* < 0.01, ^∗∗^ *p* < 0.05, and ^∗∗∗^ *p* < 0.001.

Because our data suggest a novel role for TcES in attenuating the proinflammatory response of LPS-induced BMDM, and the current M1/M2 paradigm scarcely describes the influence of TcES in the transcriptional profile of macrophages, we performed a proinflammatory mRNA array screen (see “mRNA array” for details) on M_0_, M_LPS_, M_TcES_, and M_TcES+LPS_, at 4 and 24 h poststimulus (Figure 3). Commonly produced mRNAs among the groups of M_LPS_, M_TcES_, and M_TcES+LPS_ are displayed in Table S2. As expected, our results indicate increased levels of multiple proinflammatory mRNAs in M_LPS_ with respect to M_0_ (Table 1 and Table S3), including *Il1a*, *Il6*, *Il12a*, *Il12b*, *Tnf*, and *Nos2*, among other mRNAs, at 4 and 24 h poststimulus. These molecules correspond to previously reported markers for LPS-stimulated macrophages [1]. In contrast, M_TcES_ downregulated the levels, with respect to M_0_, of cytokines, chemokines, and transcriptional factors distinctive of M1-activated macrophages, while displaying upregulated levels mainly associated with enzymes, as MAPK pathway, at 4 and 24 h poststimulus (Table 1 and Table S4). Noticeably, although M_TcES+LPS_ presented 132 and 96 upregulated mRNAs (Table S5), these macrophages only shared 6 and 3 upregulated mRNAs with M_TcES_ at 4 and 24 h poststimulus, respectively. However, M_TcES+LPS_ shared 89 and 65 upregulated mRNAs with M_LPS_ at 4 and 24 h poststimulus, respectively, including transcripts for cytokines, chemokines, receptors, and transcriptional factors as *Il1a*, *Il1b*, *Il6*, *Il12a*, *Il12b*, *Ccl3*, *Ccl5*, *Ccl2*, *Ccl7*, *Cd86*, *Tlr2*, *Stat1*, *Stat3*, and *Nfkb1* mRNA. The differentially induced mRNAs between M_TcES+LPS_ and M_LPS_ are shown in Table S6. Next, we validated 7 mRNAs associated with M1 (*Il1b*, *Stat1*, *Cd86*, *Il6*, and *Il12b*) and M2 (*Stat6* and *Chi3l3*) macrophages by RT-qPCR. The levels of these mRNAs were comparable to those observed in the mRNA array (Figure 4), which attest for the high quality of our array, supporting that a posttranscriptional mechanism induced by TcES may have a role in macrophage's response to LPS. Interestingly, although the levels of IL-6 and IL-12 in supernatants from M_TcES+LPS_ were significantly reduced respect to M_LPS_ (Figures 1(a) and 1(b)), the levels of their mRNAs of these cytokines were comparable between M_TcES+LPS_ and M_LPS_. These data suggest that posttranscriptional mechanisms triggered by TcES may have a role in modulating the production of specific inflammatory cytokines.

### 3.3. TcES Modulate the Profile of MicroRNAs in LPS-Stimulated BMDM

**Figure 5:**
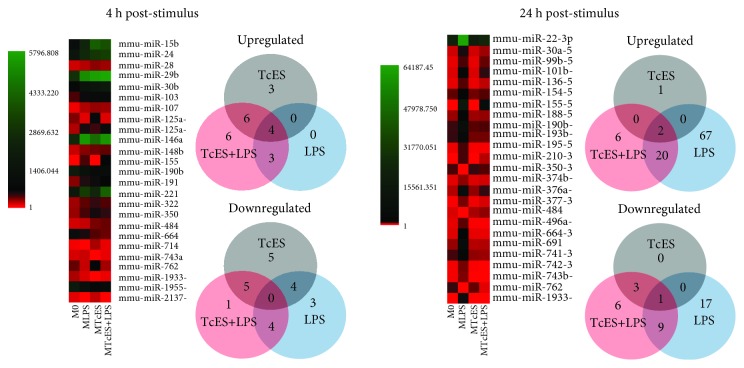
Differentially and commonly produced microRNAs in stimulated BMDM. The heat map shows the top 25 of microRNAs produced among M_LPS_, M_TcES_, and M_TcES+LPS_ 4 h and 24 h poststimulus. Each row represents a microRNA, and each column represents a specific sample. The color scale illustrates the relative level of microRNAs: green, increased production; red, decreased production; and black, mean value. Venn diagram showing the unique and overlapping microRNAs. A total of 22 and 96 modified microRNAs were found at 4 and 24 h poststimulation.

MicroRNAs participate in diverse biological processes at the posttranscriptional regulatory level. The complementary binding of microRNAs to mRNAs reduces either transcription or translation of mRNA transcripts [16]. Recently, a handful of studies indicate a role for helminth parasites and their antigens in inducing microRNAs to modulate host immune responses [14, 15, 23]. To determine whether the ability of TcES in attenuating the inflammatory response of BMDM is associated with the production of specific microRNAs, we performed a microRNA array (see “microRNA array” in Materials and Methods for details) in M_0_, M_LPS_, M_TcES_, and M_TcES+LPS_, at 4 and 24 h poststimulus. As a result, we identified 7 and 89 upregulated microRNAs in M_LPS_ at 4 h and 24 h, respectively. M_TcES_ displayed 13 (4 h) and 3 (24 h), and M_TcES+LPS_ showed 19 (4 h) and 28 (24 h) upregulated microRNAs (Figure 5). The top 10 up- and downregulated microRNAs in M_LPS_, M_TcES_, and M_TcES+LPS_ are shown in Table 2. The complete lists of microRNAs are shown in Table S7–S9. Additionally, we found 4 and 2 microRNAs shared among the groups of stimulated BMDM at 4 and 24 h, respectively (Table S10). Interestingly, M_TcES+LPS_ shared 6 upregulated microRNAs with M_TcES_ and only 3 with M_LPS_ at 4 h poststimulus. However, M_TcES+LPS_ did not share microRNAs with M_TcES_ and shared 22 with M_LPS_ at 24 h poststimulus. Finally, M_TcES+LPS_ differentially induced 3 and 20 microRNAs compared to M_LPS_ at 4 and 24 h poststimulation, respectively (Table S11). These data suggest that TcES induce the early production (4 h) of microRNAs, followed by the stimulus with LPS (24 h), in M_TcES+LPS_. This phenomenon is associated with an increased number of upregulated microRNAs in M_TcES_ compared to M_LPS_ (13 vs. 7 microRNAs, Figure 5) at 4 h poststimulation.

**Figure 6:**
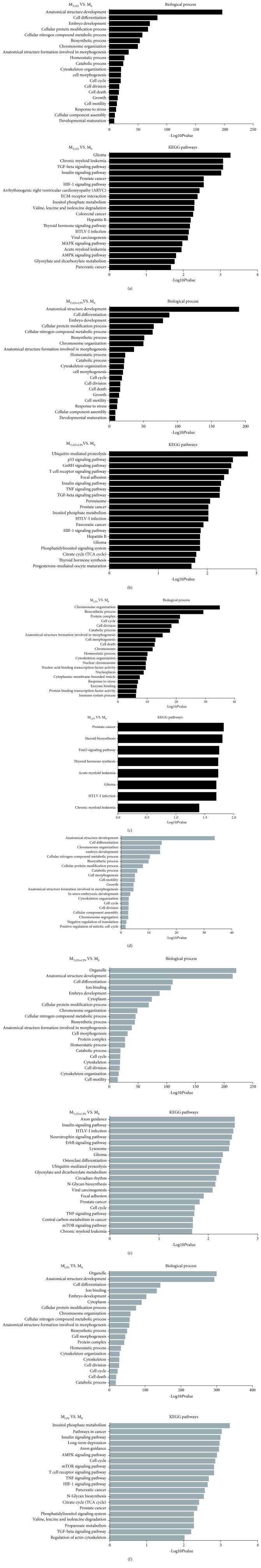
Top 20 of GO biological processes and KEGG pathways in M_TcES_, M_TcES+LPS_, and M_LPS_ at 4 (black) and 24 h poststimulus (gray). GO biological processes and KEGG pathways enriched by the upregulated differentially produced microRNAs between M_TcES_ vs. M_0_ (a and d), M_TcES+LPS_ vs. M_0_ (b and e), and M_LPS_ vs. M_0_ (c and f) at 4 h and 24 h poststimulus, respectively.

To assess the potential biological relevance of the upregulated microRNAs in the transcriptional profile of activated macrophages, we conducted bioinformatic analysis as GO terms and KEGG pathway analysis by comparing M_TcES_, M_TcES+LPS_, or M_LPS_ vs. M_0_ at both 4 and 24 h poststimulus. The GO terms in M_TcES_ and M_TcES+LPS_ were mainly enriched for the biological process associated with anatomical structure development, cell differentiation, and cellular protein differentiation process at 4 h poststimulus (Figures 6(a) and 6(b)). Anatomical structure development, cell differentiation, and chromosome organization were predicted to be a target by microRNAs in M_TcES_, while organelle, anatomical structure, and cell differentiation were enriched in M_TcES+LPS_ at 24 h poststimulus (Figures 6(d) and 6(e)). Lastly, GO terms enriched for M_LPS_ are chromosome organization, biosynthetic process, and protein complex as well as organelle, anatomical structure, and cell differentiation at 4 and 24 h poststimulus, respectively (Figures 6(c) and 6(f)). The KEGG pathway enrichment analysis revealed that at 4 h stimulus, upregulated microRNAs were regulating glioma, chronic myeloid leukemia, and TGF-*β* signaling pathway in M_TcES_ (Figure 6(a)); ubiquitin-mediated proteolysis, p53, and GhRH signaling pathway in M_TcES+LPS_ (Figure 6(b)); and prostate cancer, steroid biosynthesis, and FoxO signaling pathway in M_LPS_ (Figure 6(c)). In contrast, the KEGG enrichment pathways at 24 h poststimulus were axon guidance, insulin signaling pathway, and HTLV-I infection in M_TcES+LPS_ (Figure 6(e)) and inositol phosphate metabolism, pathways in cancer, and insulin signaling pathway in M_LPS_ (Figure 6(f)). For more details of GO enrichment analysis and KEGG pathways, refer to Table S12 and Table S13. These data suggest a role for TcES in inducing microRNAs that regulate important metabolic, cell signaling, and inflammatory pathways in LPS-stimulated BMDM.

**Figure 7:**
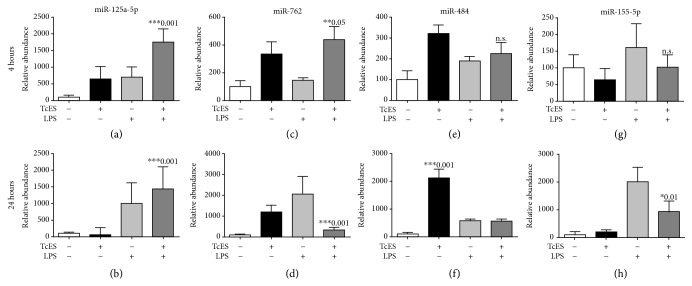
Validation and identification of microRNAs in stimulated BMDM. Macrophages were cultured in the presence of TcES (25 *μ*g/mL), LPS (1 *μ*g/mL), or a combination of TcES+LPS for 4 (left bar) and 24 h poststimulus (right bar). Relative levels of selected microRNAs were determined by TaqMan miRNA assays after normalization with 18S RNA. MicroRNA levels are represented as fold change relative to PBS-treated BMDM (FC ± SD). Data shown are representative of two independent experiments. Significance was calculated using *t*-test. ^∗^ *p* < 0.01, ^∗∗^ *p* < 0.05, and ^∗∗∗^ *p* < 0.001.

Next, we selected and validated by RT-qPCR four microRNAs (miR-125a-5p, miR-762, miR-155-5p, and miR-484), which are potentially involved in the regulation of inflammatory mRNAs, as indicated by previous studies and our bioinformatics analysis. We found that both M_LPS_ and M_TcES_ showed increased levels of miR-125a-5p (Figure 7(a)), a microRNA reported to reduce the production of inflammatory cytokines (IL-6, IL-12, and TNF*α*) [24]. The levels of miR-125a-5p were sustained in M_LPS_ and M_TcES+LPS_ until 24 h poststimulus (Figure 7(b)). The combined stimuli of TcES and LPS induced an additive effect in the levels of this microRNA at 4 h poststimulation (Figure 7(a)). Furthermore, M_TcES_ and M_TcES+LPS_ showed increased levels of miR-762, a microRNA known to directly target the mRNA of inflammatory transcription factor *Irf7* [25], at 4 h poststimulus (Figure 7(c)). This microRNA was later produced in M_LPS_ at 24 h poststimulation (Figure 7(d)). In addition, miR-484 was highly produced in M_TcES_ compared to both M_TcES+LPS_ and M_LPS_ at 4 and 24 h poststimulus (Figures 7(e)–7(f)). Our bioinformatic analysis suggests that miR-484 can potentially target *Nfkb*, *Stat5a*, *Irf1*, *Myd88*, *Stat1*, and *Il12a* mRNAs. Finally, miR-155-5p, a well-defined microRNA in M1 macrophages, was upregulated in M_LPS_ and M_TcES+PS_ compared to M_TcES_ at 4 and 24 h poststimulation (Figures 7(g)–7(h)). The profile of these miRNAs was comparable to those observed in the microRNA array. Altogether, our findings suggest a role for miR-125a-5p, miR-762, and miR-484 in the immunomodulatory effect of TcES in BMDM.

## 4. Discussion

Helminth parasites and their antigens can counteract proinflammatory responses generated during autoimmune diseases [3]. In our laboratory, we have previously demonstrated that infection with the helminth parasite *T. crassiceps* or the administration of TcES reduced the symptoms of EAE, type I diabetes, and ulcerative colitis, in part due to the polarization of macrophages *in vivo* towards an M2 phenotype [4–8, 26]. However, the functional role of TcES in regulating the activation and inflammatory response of macrophages remains unknown. In this study, we evaluated the effect of TcES on the polarization towards an M2 profile, inflammatory immune response, and transcriptomic profile of macrophages *in vitro*.

We first measure the production of the cytokines IL-6, IL-10, IL-12, and TNF*α* in BMDM-stimulated with TcES alone or in combination with LPS and observed that TcES increased the levels of the regulatory cytokine IL-10 and reduced the release of the inflammatory cytokines IL-6, IL-12, and TNF*α* in supernatants from LPS-stimulated BMDM. TcES alone did not increase the production of inflammatory cytokines but induced the release of IL-10 in BMDM. The levels of both mRNAs of *Il10* and *Tnf* measured by RT-qPCR showed similar trends when compared to the levels of cytokines obtained by ELISA assay, suggesting a consistent role for TcES in regulating cytokine production by inhibition of their transcripts.

Here, we evaluated the production of NOS2 and ARG1 in BMDM stimulated with TcES alone or in combination with IL-4 or LPS. M1 macrophages normally produce NOS2, which metabolizes L-arginine to nitric oxide (NO), while M2 macrophages produce ARG1, which metabolizes L-arginine to produce prolines and polyamines [2, 27]. We found that whereas BMDM stimulated with IL-4 or LPS alone showed increased levels of ARG1 and NOS2, respectively, TcES did not alter the production of both NOS2 and ARG1, after 24 h poststimulation. Our data are in agreement with previous studies using *Fasciola hepatica* tegumental antigens, which also failed to directly induce the production of molecules associated with M2 macrophages *in vitro* but not *in vivo* [28]. The production of M2 canonical molecules such as ARG1 has been reported to be IL-4-dependent, which is produced by T_H_2 T cells, natural killer T cells, and basophils but not macrophages [29–31]. Therefore, helminth antigen stimulation alone is not enough to induce functional polarization of BMDM towards M2; however, they influence the inflammatory properties of these cells. Therefore, TcES do not induce the production of M2-associated molecules but counteract inflammatory response in macrophages *in vitro*.

Recent studies indicate a regulatory role for helminth antigens obtained from *Trichinella spiralis*, *Spirometra erinaceieuropaei*, *Schistosoma mansoni*, and *Hymenolepis diminuta* in reducing cytokine production and subsequent inflammation [32–41]. However, the analysis of a small number of inflammatory products and/or conventional M1 and M2 markers poorly describes the effect of these antigens in the proinflammatory profile of macrophages. Therefore, using array approaches (nCounter Inflammation Panel, NanoString mRNAs), we determined the levels of multiple mRNAs involved in macrophage inflammatory response. As expected, M_TcES_ displayed a lower number of upregulated inflammatory mRNAs, when compared to M_LPS_ at 4 (42 vs. 120 mRNAs) and 24 h poststimulus (36 vs. 104 mRNAs). M_TcES_ induced mRNAs associated with phagocytosis, M2 macrophage, and anti-inflammatory response. For instance, M_TcES_ showed increased levels of *Pkca* mRNA necessary for the biogenesis of phagolysosomes [42]. In addition, M_TcES_ increased levels of *Irf3*, *C1s*, and *Ptgs* mRNAs which have been previously associated with anti-inflammatory microenvironments and identified in M2 macrophages [43–53]. Although our results suggest that TcES induce mRNAs associated with M2 macrophages, the stimulus with these helminth-derived molecules is not enough to induce a full expression of all M2 markers in macrophages as observed in previously reported studies [28].

In contrast, M_TcES+LPS_ and M_LPS_ shared more than 60 proinflammatory mRNAs at both 4 and 24 h poststimulation. Interestingly, we observed reduced levels of different inflammatory mRNAs, e.g., *Nox1*, *Ccl21a*, *Ccr4*, and *Cxcr2*, in M_TcES+LPS_ with respect to M_LPS_ at 24 h poststimulus. Noteworthily, although the levels of *Il6*, *Il12a*, and *Il12b* mRNAs were similar between M_TcES+LPS_ and M_LPS_, reduced levels of these cytokines were detected in supernatants from M_TcES+LPS_ with respect to M_LPS_. A similar phenomenon has been reported for *Acanthocheilonema viteae* antigens, in decreasing TNF*α* production in macrophages without altering *Tnf* transcripts [35], suggesting the participation of other posttranscriptional mechanisms. Additionally, M_TcES_ and M_TcES+LPS_ shared levels of the mRNAs for *Irf3*, *Defa1*, *C1s1*, and *Ifna1* at 4 h, and Hspb1, Maff, and Map2k6 at 24 h post stimulus. While levels of *Irf3* and *C1s1* mRNAs suggest an M2-like profile, levels of *Ifna1* mRNA suggest that TcES could be recognized through TLR3, TLR7/8, or TLR9 [54, 55]. To note, *Defa1* mRNA codifies protein HNP1 (human neutrophil-*α* defensin), which inhibits macrophage-driven inflammation through targeting proinflammatory cytokines and NO [56, 57]. Lastly, we noted that *Tlr2* mRNA was upregulated in M_TcES+LPS_ at 24 h post stimulus, which could be attributed to TcES's own recognition, as previously have been reported to recognize TcES [58]. These data suggest that posttranscriptional events may be involved in the regulatory mechanism triggered by TcES in regulating macrophage inflammatory responses.

microRNAs, small noncoding RNA molecules, have emerged as a key component of macrophage posttranscriptional regulation [59]. These molecules can silence the translation of mRNAs via base-pairing with complementary sequences within the RNA molecules. Hence, we further analyzed the microRNA profile in BMDM stimulated with TcES alone or in combination with LPS. Our analysis demonstrated that M_TcES+LPS_ shared regulatory microRNAs with M_LPS_. For example, miR-146a-5p was upregulated in M_TcES+LPS_ and M_LPS_ at 4 h and only in M_TcES+LPS_ at 24 h poststimulus. This microRNA has been reported to dampen proinflammatory responses in macrophages through the inhibition of TLRs, NF-*κ*B, and STAT signaling pathways by targeting the mRNAs of *Traf6*, *Irak1*, *Irak2*, *Nfκb*, *Stat1*, and *Ap1* [60–63]. This evidence is supported by our KEGG enrichment analysis, which indicates that overproduced microRNAs in M_TcES+LPS_ target mRNAs involved in NF-*κ*B, TNF, and MAPK signaling pathways. Of note, these data also confirm our hypothesis that TcES target proinflammatory pathways and support our previous findings indicating a role for TcES in blocking the IFN-*γ*/STAT1 signaling pathway in macrophages *in vitro* [17].

M_TcES+LPS_ also overproduced microRNAs previously reported to target inflammatory mRNAs; for instance, let-7i and let-7e target *Tlr4* mRNA, which causes a drop in the recognition of proinflammatory antigens [64–66]. Moreover, miR-24-3p production in macrophages has been reported to significantly decrease the production of IL-6 and TNF*α* [67]. Furthermore, M_TcES+LPS_ and M_TcES_ shared upregulated microRNAs previously reported to be elicited in macrophages exposed to *E. multilocularis* antigens (e.g., miR-146a-5p) and *S. japonicum* (miR-365 and miR-24) [14, 68]. These data suggest the presence of conserved antigens among helminths that could trigger similar posttranscriptional mechanisms to modulate immune responses in the host.

Finally, we selected four upregulated microRNAs to validate their levels by RT-qPCR and confirm the high quality of our array. We observed increased levels of miR-125a-5p in M_TcES_ and M_LPS_, as early as 4 h poststimulus. The combined stimulus with TcES and LPS induced an additive effect in the levels of this microRNA. miR-125a-5p has been reported to increase after TLR2/4 signaling and has a key role in reducing the production of inflammatory cytokines (IL-6, IL-12, and TNF*α*) by targeting NF-*κ*B and KFL4 signaling pathways [24, 69–71]. These data are associated with our previous studies suggesting that TcES is a ligand of TLR2 in phagocytic cells [58, 72]. In addition, miR-762 was selectively induced in M_TcES_ and M_TcES+LPS_ at 4 h poststimulus. miR-762 has been demonstrated to increase in ovarian and breast cancer and ocular tissue [73–75] where macrophages normally acquire an M2-like phenotype [76, 77]. Furthermore, by using bioinformatic tools, we found *Il12b*, *Il6, Tnf*, *Nfkb*, and *Cd86* mRNAs as possible targets of miR-762 in M_TcES_ and M_TcES+LPS_. The microRNA miR-484 was found to be upregulated in all the groups of stimulated BMDM at 4 h; however, its levels were only sustained in M_TcES_ at 24 h poststimulation. miR-484 has been previously identified in multiple types of cancers [78–82] and the cerebral cortex [83]; such microenvironments are known to promote an anti-inflammatory phenotype in macrophages. Our bioinformatic analysis shows that *Il1b*, *Nfkb*, *Stat5a*, *Irf1*, *Myd88*, *Stat1*, and *IL-12a* mRNAs are possible targets for miR-484, which suggest a possible role for miR-484 in immune tolerance.

Lastly, we observed that miR-155-5p was upregulated in M_LPS_ at 4 h and M_TcES+LPS_ and M_LPS_ at 24 h poststimulus. miR-155-5p is a well-defined microRNA induced by LPS in macrophages, which enhances the proinflammatory response by targeting the immunomodulatory mRNAs *Ship1*, *Socs1*, *Il13rα*, and *C/ebpβ* and increasing the half-life of *Tnf* [64, 84–88]. However, antigens of another helminth, *Angiostrongylus cantonensis*, also upregulated miR-155-5p [89]. Therefore, it would be of interest to further study the role of miR-155-5p during exposure to helminth antigens.

In summary, our study demonstrates a role for TcES in regulating the production of key inflammatory cytokines, possibly by inducing microRNAs that target inflammatory transcripts and promoting the release of IL-10 in macrophages. This phenomenon shapes the transcriptomic profile of macrophages and consequently the outcome of the immune response. Although we found clear associations between TcES-induced microRNAs and mRNAs involved in multiple inflammatory pathways as their targets, our study has the limitation that we did not prove a direct interaction between microRNAs and mRNAs. Therefore, future studies in our laboratory will focus on elucidating the functional roles and significance of the different microRNAs described here. These findings increase our understanding of how released molecules from helminths regulate inflammation and may offer new approaches for the treatment of autoimmune and inflammatory diseases.

## Figures and Tables

**(a):** 

4 h poststimulus											
MTcES vs. M0				MTcES+LPS vs. M0				MLPS vs. M0			
Upregulated		Downregulated		Upregulated		Downregulated		Upregulated		Downregulated	
mRNAs	FC	mRNAs	FC	mRNAs	FC	mRNAs	FC	mRNAs	FC	mRNAs	FC
Cytokines		Cytokines		Cytokines		Receptors		Cytokines		Cytokines	
*Ifna1*	2.8	*Il12b*	0.2	*Il6*	5974.7	*Ccr2*	0.2	*Il6*	5140.9	*Ifna1*	0.2
Chemokines		*Il23a*	0.2	*Il1a*	3556.5	*Cd163*	0.1	*Il1a*	4198.0	Transcriptional factors	
*Ccl21a*	6.3	*Il1b*	0.1	*Il12a*	626.2	*Ptger3*	0.1	*Il12a*	739.4	*Mef2c*	0.2
Transcriptional factors		Complement proteins		*Il12b*	582.3	*Ccr3*	0.1	*Il12b*	957.3	Receptors	
*Irf3*	8.8	*C2*	0.2	Chemokines		Transcriptional factors		Chemokines		*Ptger3*	0.1
*Mafg*	3.1	Receptors		*Cxcl3*	2527.8	*Mef2c*	0.2	*Cxcl3*	2285.7	*Ccr2*	0.2
Complement proteins		*Cxcr1*	0.2	*Cxcl10*	2060.0	Enzymes		*Cxcl10*	2215.6	*Cd163*	0.1
*C1s*	6.3	Transcriptional factors		*Cxcl9*	1285.0	*Limk1*	0.4	*Cxcl9*	1699.8	Enzymes	
Antiviral proteins		*Elk1*	0.2	*Ccl22*	666.9			*Ccl22*	540.2	*Plcb1*	0.5
*Hspb1*	4.5	Inflammatory-related proteins		Inflammatory-related proteins				Inflammatory-related proteins		*Ptgs1*	0.4
Enzymes		*Lta*	0.2	*Ptgs2*	6106.5			*Ptgs2*	5432.4	*Limk1*	0.3
*Defa-rs1*	8.0	Enzymes		*Oasl1*	570.6			*Oasl1*	504.8		
*Prkca*	8.0	*Flt1*	0.2								
*Ptgs1*	2.1	*Plcb1*	0.1								
Others		Others									
*Mbl2*	4.5	*Kng1*	0.4								

FC: fold change.

**(b):** 

24 h poststimulus											
MTcES vs. M0				MTcES+LPS vs. M0				MLPS vs. M0			
Upregulated		Downregulated		Upregulated		Downregulated		Upregulated		Downregulated	
mRNAs	FC	mRNAs	FC	mRNAs	FC	mRNAs	FC	mRNAs	FC	mRNAs	FC
Cytokines		Cytokines		Cytokines		Cytokines		Cytokines		Cytokines	
*Ifna1*	3.7	*Il1b*	0.4	*Il12a*	338.8	*Tgfb3*	0.2	*Il12a*	252.8	*Tgfb3*	0.2
Chemokines		Chemokines		*Il6*	194.2	Receptors		*Il6*	165.3	Receptors	
*Ccl21a*	6.4	*Cxcl10*	0.0	Chemokines		*Ccr3*	0.1	Chemokines		*Mrc1*	0.0
Antiviral proteins		*Ccl24*	0.3	*Ccl5*	2687.8	*Cd163*	0.2	*Ccl5*	3007.4	*Cd163*	0.1
*Hspb1*	4.8	*Cxcl2*	0.4	*Cxcl3*	1206.0	*Mrc1*	0.0	*Cxcl3*	823.1	*Trem2*	0.2
Enzymes		Receptors		*Cxcl9*	410.0	*Trem2*	0.2	*Cxcl9*	332.7	*Ccr3*	0.4
*Alox5*	8.9	*Tlr2*	0.2	Enzymes		Enzymes		Complement-related proteins		Enzymes	
*Plcb1*	5.2	*Cd86*	0.3	*Ptgs2*	206.4	*Ptgs1*	0.0	*Cfb*	17948.5	*Prgs1*	0.1
*Prkca*	12.1	Transcriptional factors		Receptors		Transcriptional factors		Receptors		Transcriptional factors	
*Map2k6*	5.3	*Cebpb*	0.4	*Il23r*	206.8	*Myc*	0.2	*Cxcr1*	175.2	*Maff*	0.2
*Ppp1r12b*	4.0	*Relb*	0.5	*Cxcr1*	175.7	*Mef2c*	0.4	*Il23r*	215.8	*Myc*	0.1
*Defa-s1*	3.1	Enzymes		*Cd40*	155.6			*Cd40*	128.3	*Mef2c*	0.4
*Map3k9*	3.1	*Nos2*	0.3	Complement-related proteins				Enzymes			
		Antiviral proteins		*Cfb*	19872.2			*Ptgs2*	144.5		
		*Ifit1*	0.3	Inflammatory-related proteins							
				*Areg*	2.5						

FC: fold change.

**(a):** 

4 h poststimulus											
MTcES vs. M0				MTcES+LPS vs. M0				MLPS vs. M0			
Upregulated		Downregulated		Upregulated		Downregulated		Upregulated		Downregulated	
MicroRNA	FC	MicroRNA	FC	MicroRNA	FC	MicroRNA	FC	MicroRNA	FC	MicroRNA	FC
mmu-miR-421-3p	102.60	mmu-miR-539-5p	0.02	mmu-miR-155-5p	676.18	mmu-miR-190b-5p	0.49	mmu-miR-155-5p	402.62	mmu-miR-125a-3p	0.33
mmu-miR-484	2.35	mmu-miR-467c-5p	0.02	mmu-miR-546	112.84	mmu-miR-1955-5p	0.47	mmu-miR-546	76.67	mmu-miR-1224-5p	0.31
mmu-miR-350-3p	2.22	mmu-miR-1193-3p	0.02	mmu-miR-128-3p	88.90	mmu-miR-1933-5p	0.41	mmu-miR-146a-5p	2.09	mmu-miR-467f	0.02
mmu-miR-148b-3p	2.14	mmu-miR-199a-3p	0.01	mmu-miR-421-3p	84.34	mmu-miR-125a-3p	0.36			mmu-miR-539-5p	0.02
mmu-miR-125a-3p	2.13	mmu-miR-383-5p	0.01	mmu-miR-331-3p	74.08	mmu-miR-664-3p	0.35			mmu-miR-380-3p	0.02
mmu-miR-191-5p	2.12	mmu-miR-489-3p	0.01	mmu-miR-484	2.62	mmu-miR-1224-5p	0.35			mmu-miR-467c-5p	0.02
mmu-miR-30b-5p	2.09	mmu-miR-1953	0.01	mmu-miR-191-5p	2.46	mmu-miR-1193-3p	0.02			mmu-miR-714	0.02
mmu-miR-103-3p	2.07	mmu-miR-743a-3p	0.01	mmu-miR-30b-5p	2.44	mmu-miR-2137	0.01			mmu-miR-489-3p	0.01
mmu-miR-29b-3p	1.99	mmu-miR-410-3p	0.01	mmu-miR-99b-5p	2.40	mmu-miR-210-3p	0.01			mmu-miR-2137	0.01
		mmu-miR-1933-5p	0.00	mmu-miR-148b-3p	2.32	mmu-miR-804	0.01			mmu-miR-804	0.01

FC: fold change

**(b):** 

24 h poststimulus											
MTcES vs. M0				MTcES+LPS vs. M0				MLPS vs. M0			
Upregulated		Downregulated		Upregulated		Downregulated		Upregulated		Downregulated	
MicroRNA	FC	MicroRNA	FC	MicroRNA	FC	MicroRNA	FC	MicroRNA	FC	MicroRNA	FC
mmu-miR-362-5p	77.49	mmu-miR-743b-5p	0.01	mmu-miR-155-5p	4272.7	mmu-miR-326-3p	0.48	mmu-miR-155-5p	14139.0	mmu-miR-2137	0.01
mmu-miR-421-3p	93.67	mmu-miR-1949	0.01	mmu-miR-210-3p	285.39	mmu-miR-361-5p	0.34	mmu-miR-1933-5p	901.35	mmu-miR-362-3p	0.01
mmu-miR-1929-5p	74.93	mmu-miR-2134	0.01	mmu-miR-674-5p	171.63	mmu-miR-27a-3p	0.32	mmu-miR-210-3p	639.8	mmu-miR-484	0.01
		mmu-miR-2137	0.01	mmu-miR-331-3p	127.7	mmu-miR-221-3p	0.29	mmu-miR-574-3p	579.11	mmu-miR-152-3p	0.01
				mmu-miR-7a-5p	109.76	mmu-miR-23a-3p	0.26	mmu-miR-673-3p	518.4	mmu-miR-714	0.005
				mmu-miR-574-3p	96.8	mmu-miR-27b-3p	0.26	mmu-miR-674-5p	331.59	mmu-miR-107-3p	0.004
				mmu-miR-467a-5p	92.80	mmu-miR-762	0.23	mmu-miR-466a-5p	308.23	mmu-miR-324-5p	0.004
				mmu-miR-1929-5p	88.81	mmu-miR-1224-5p	0.22	mmu-miR-489-3p	284.88	mmu-miR-148b-3p	0.003
				mmu-miR-489-3p	87.81	mmu-miR-199a-3p	0.01	mmu-miR-1900	261.532095	mmu-miR-350-3p	0.002
				mmu-miR-139-5p	83.82	mmu-miR-145-5p	0.01	mmu-miR-1953	242.8512311	mmu-miR-762	0.001

FC: fold change.

## Data Availability

The array data used to support the findings of this study have been deposited in the GEO (Gene Expression Omnibus) database of the NCBI with the accession numbers GSE125170 for RNAm and GSE125171 for microRNA as part of the SuperSerie GSE125172 which are public once this article is published.

## Abbreviations

M2: Alternatively activated-like macrophages
Arg1: Arginase 1
EAE: Autoimmune encephalomyelitis
BMDM: Bone marrow-derived macrophages
M1: Classical activation in macrophages
DAMPs: Danger-associated molecular patterns
DMEM: Dulbecco's modified Eagle's media
FBS: Fetal bovine serum
FC: Fold change (FC)
Fizz1: Found in inflammatory zone
GO: Gene ontology
HNP1: Human neutrophil-*α* defensin
IL-4R*α*: IL-4 receptor *α*
IGFBP5: Insulin-like growth factor binding protein 5
IL-10: Interleukin-10
IL-12: Interleukin-12
IL-4: Interleukin-4
IL-6: Interleukin-6
KEGG: Kyoto Encyclopedia of Genes and Genomes
LPS: Lipopolysaccharide
M-CSF: Macrophage colony-stimulating factor
MHC: Major histocompatibility complex
MR: Mannose receptor
mRNAs: Messenger RNAs
NO: Nitric oxide
Nos2: Nitric oxide synthase
PAMPs: Pathogen-associated molecular patterns
PD-L2: Programmed death ligand 2
TcES: *Taenia crassiceps*-excreted/secreted antigens
TLR: Toll-like receptor
TNF*α*: Tumor necrosis factor *α*
UTR: Untranslated region.

## Supplementary Materials

Supplementary Materials Table S1: sequence of primers used in RT-qPCRs to amplify the specified mRNAs (A) or microRNAs (B). Table S2: mRNAs commonly upregulated (≥2-fold change) in BMDM with TcES (MTcES), LPS (MLPS), or TcES+LPS (MTcES+LPS) at both 4 (left) or 24 h (right) poststimulus. Table S3: transcripts up (A) or downregulated (B) at 4 or 24 h poststimulus with LPS (MLPS). Table S4: transcripts up (A) or downregulated (B) at 4 or 24 h poststimulus with TcES (MTcES). Table S5: transcripts up- (A) or downregulated (B) at 4 or 24 h poststimulus with TcES+LPS (MTcES+LPS). Table S6: mRNAs expressed differently (≥2 FC) between BMDM stimulated with TcES+LPS (MTcES+LPS) and with LPS (MLPS) at 4 (left) or 24 h (right). Table S7: microRNAs up- (A) or downregulated (B) at 4 or 24 h poststimulus with LPS (MLPS). Table S8: microRNAs up- (A) or downregulated (B) at 4 or 24 h poststimulus with TcES (MTcES). Table S9: microRNAs up- (A) or downregulated (B) at 4 (left) or 24 h (right) poststimulus with TcES+LPS (MTcES+LPS). Table S10: microRNAs upregulated (≥2-fold change) in BMDM with TcES (MTcES), LPS (MLPS), and TcES+LPS (MTcES+LPS) at both 4 (left) or 24 h (right) poststimulus. Table S11: microRNAs expressed differently (≥2 FC) between BMDM with TcES+LPS (MTcES+LPS) and with LPS (MLPS) at 4 (left) or 24 h (right) poststimulus. Table S12: KEGG pathway analysis. Significantly enriched KEGG pathways (*p* < 0.05) are presented for BMDM with TcES (MTcES), TcES+LPS (MTcES+LPS), or LPS (MLPS) at 4 (left) or 24 h (right) poststimulus. Table S13: GO biological process annotations of the target genes of differentially expressed microRNAs. According to *p* value, GO terms of biological process are shown in BMDM with TcES (MTcES), TcES+LPS (MTcES+LPS), or LPS (MLPS) at 4 (left) or 24 h (right) poststimulus.
